# Autophagy and Its Impact on Neurodegenerative Diseases: New Roles for TDP-43 and C9orf72

**DOI:** 10.3389/fnmol.2017.00170

**Published:** 2017-05-30

**Authors:** Mauricio Budini, Emanuele Buratti, Eugenia Morselli, Alfredo Criollo

**Affiliations:** ^1^Dentistry Faculty, Institute in Dentistry Sciences, University of ChileSantiago, Chile; ^2^International Centre for Genetic Engineering and BiotechnologyTrieste, Italy; ^3^Department of Physiology, Faculty of Biological Sciences, Pontificia Universidad Católica de ChileSantiago, Chile; ^4^Advanced Center for Chronic DiseasesSantiago, Chile

**Keywords:** autophagy, neurodegenerative diseases, TDP-43, C9orf72, ALS, FTLD

## Abstract

Autophagy is a catabolic mechanism where intracellular material is degraded by vesicular structures called autophagolysosomes. Autophagy is necessary to maintain the normal function of the central nervous system (CNS), avoiding the accumulation of misfolded and aggregated proteins. Consistently, impaired autophagy has been associated with the pathogenesis of various neurodegenerative diseases. The proteins TAR DNA-binding protein-43 (TDP-43), which regulates RNA processing at different levels, and chromosome 9 open reading frame 72 (C9orf72), probably involved in membrane trafficking, are crucial in the development of neurodegenerative diseases such as Amyotrophic lateral sclerosis (ALS) and Frontotemporal Lobar Degeneration (FTLD). Additionally, recent studies have identified a role for these proteins in the control of autophagy. In this manuscript, we review what is known regarding the autophagic mechanism and discuss the involvement of TDP-43 and C9orf72 in autophagy and their impact on neurodegenerative diseases.

## Introduction

The population is aging worldwide. Indeed, between 2015 and 2030, the number of people in the world aged 60 years or over is projected to grow by 56 per cent, from 901 million to 1.4 billion; by 2050, the global population of older persons is projected to more than double in size, reaching nearly 2.1 billion^1^ As people age, the risk of developing a neurodegenerative disease rises dramatically (Beghi et al., 2006; Reeve et al., 2014); therefore, the study of the molecular bases of neurological disorders has become an urgent issue (Johnson, 2015).

Neurodegenerative diseases such as Alzheimer's, Parkinson's, Prion, Huntington's, and Motor neuron diseases (MND), as well as Spinal muscular atrophy, Spinocerebellar ataxia (SCA), Frontotemporal Lobar Degeneration (FTLD), and Amyotrophic lateral sclerosis (ALS) cause progressive damage in the central and peripheral nervous system, reducing the quality of life of affected patients (do Carmo Costa and Paulson, 2013). At a biochemical level, various molecular mechanisms underlie the development of neurodegenerative diseases; however, they all lead to chronic loss of function of neurons or nerves (Ciechanover and Kwon, 2015). A characteristic feature of many of these disorders is the accumulation of irregular protein structures in the neurons, which stimulate the formation of inclusion bodies and insoluble deposits (Atkin and Paulson, 2014; Ciechanover and Kwon, 2015). These features are generally associated with an inefficient protein quality control system in the neurons, which prevents the elimination of dysfunctional proteins and promotes their accumulation inside the cells (Nijholt et al., 2011).

To date, an effective treatment for these pathologies has not been discovered; only palliative approaches to alleviate patient symptoms are currently used (Lokk and Delbari, 2012; do Carmo Costa and Paulson, 2013). Thus, it is critical to identify novel molecular targets to develop new therapies for their treatment (do Carmo Costa and Paulson, 2013).

The TAR-DNA binding protein 43, TDP-43, and the chromosome 9 open reading frame 72, C9orf72, are closely associated with the development of ALS, a progressive neurodegenerative disease that affects nerve cells in the brain and the spinal cord, and Frontotemporal Lobar Degeneration (FTLD), a form of dementia characterized by a progressive decline in behavior and/or language (Baralle et al., 2013; Cruts et al., 2013; Rohrer et al., 2015). TDP-43 is a nuclear factor involved in molecular pathways that control the fate of cellular RNAs, and, in contrast to healthy neurons, it is found in an aggregated form in neurons of ALS and FTLD patients (Arai et al., 2006; Neumann et al., 2006). The potentially toxic role of TDP-43 aggregates is poorly understood; however, it has been proposed that they might act as “sink” for newly synthetized, functional TDP-43 protein, leading to TDP-43 loss-of-function in the cell (Budini et al., 2014, 2015). Thus, these insoluble aggregates of TDP-43 probably impair the normal processing of specific RNA targets, promoting neuronal cell death and, therefore, neurodegeneration.

To date, the physiological functions C9orf72 have not been completely understood; however, recent studies suggest that C9orf72 can be involved in membrane trafficking and in autophagy (Sellier et al., 2016; Sullivan et al., 2016). For example, C9orf72 generates nuclear RNA granules that abnormally sequester key factors required for cellular homeostasis in both ALS and FTLD patients (Cruts et al., 2013).

Among the different cellular pathways that could be involved in the onset of ALS and FTLD autophagy has recently received considerable attention (Menzies et al., 2015). Together with the proteasome, autophagy has been identified as the main pathway that promotes protein degradation and, when altered, it impairs cell homeostasis and physiology (Nijholt et al., 2011). In this regard, *in vivo* and *in vitro* studies support the hypothesis that autophagy is responsible for the degradation of TDP-43 (Scotter et al., 2014). In addition, recent observations suggest that TDP-43 is not just a passive substrate for autophagy; rather, it seems actively involved in its initiation (Bose et al., 2011). Similarly, recent evidences indicate that C9orf72 appears to be a key autophagic regulator, functioning through protein-protein interactions (Webster et al., 2016).

Here, we will discuss evidence of the correlation of TDP-43 and C9orf72 with the autophagic pathway. This manuscript will help clarify how the “cross-regulation” between TDP-43 and C9orf72 impacts autophagy and, therefore, the onset and the progression of neurodegenerative diseases such as ALS and FTLD.

## Autophagy

### An overview of the autophagy process

**Figure 1:**
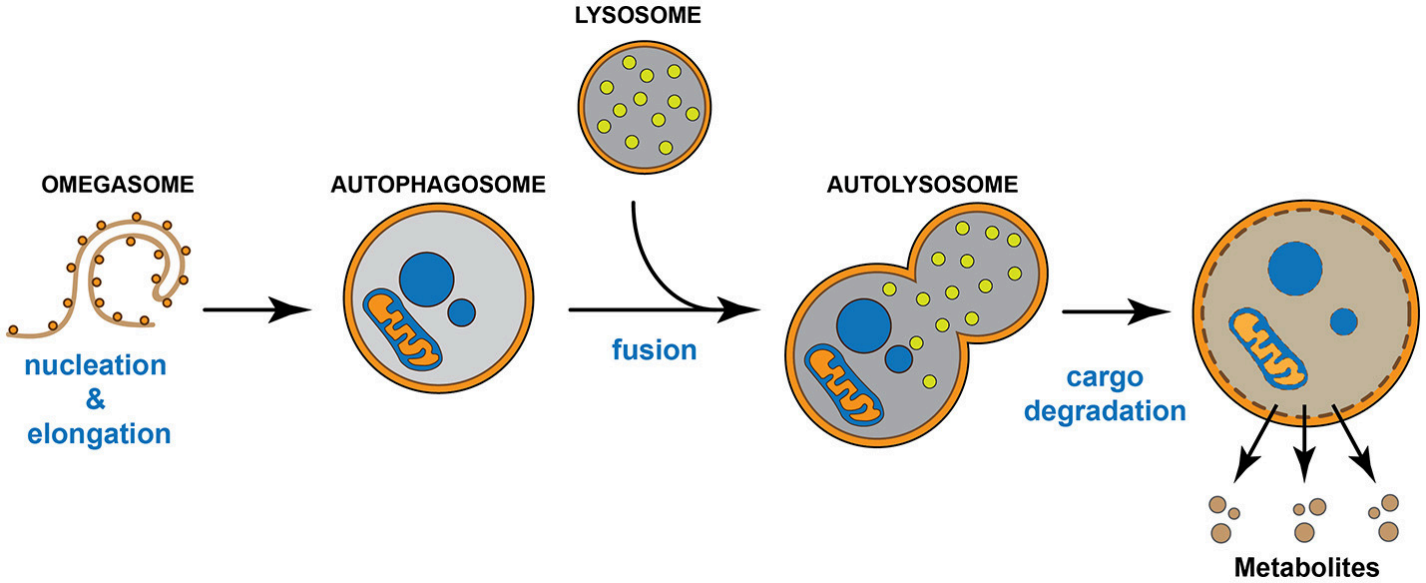
Autophagic pathway. The omegasome expands its membrane to form a double membrane organelle called autophagosome, which sequesters intracellular material such as proteins and organelles. Then, the autophagosome fuses with the lysosome to form the autolysosome. The lysosome supplies hydrolytic enzymes, which are activated following the autophagosome-lysosome fusion, promoting the degradation of the autolysosome cargo. The digestion of the cargo generates new metabolites that turn back to the cytosol and that will be re-used by the cell.

Autophagy is a regulated auto-degradative intracellular process highly conserved across many species, from yeast to mammals. Macroautophagy, hereafter referred to as “autophagy,” begins with the formation of a primitive membrane structure called the “omegasome,” which arises from intracellular membranous domains. The omegasome sequesters cytosolic targets (Lamb et al., 2013; Mizumura et al., 2014), which can include aggregates or insoluble proteins, damaged and/or oxidized organelles such as mitochondria (mitophagy), endoplasmic reticulum (ERphagy), peroxisomes (pexophagy), lipid droplets (lipophagy), and ferritin (ferritinophagy) (Iwata et al., 2006; Narendra et al., 2008; Lamb et al., 2013), and becomes known as an autophagosome. The autophagosome, in turn, fuses with a lysosome to form an “autophagolysosome,” where the cargo is degraded by hydrolytic lysosomal enzymes in either a bulk or selective manner (Lamb et al., 2013; Mizumura et al., 2014). This autophagic degradation results in the production of available metabolites that support key metabolic cellular requirements (Bento et al., 2016) (Figure 1). Nutritional stress (starvation) represents the most common condition to induce autophagy; however, other stress stimuli such as ER stress, hypoxia, mitochondrial damage, free radicals, and genotoxic stress can also induce it (Bento et al., 2016). Importantly, stress-induced autophagy and basal autophagy are crucial to maintain cellular homeostasis *in vitro* and *in vivo* (Fernandez and Lopez-Otin, 2015). Indeed, unbalanced autophagy, either increased or reduced, contributes to the progression of various human diseases, including neurodegenerative diseases, muscle disorders, cancer, metabolic dysfunctions, cardiovascular, and infectious diseases (Fernandez and Lopez-Otin, 2015).

How can autophagy be involved in the regulation of such a variety of human illnesses and disorders? As previously mentioned, autophagy functions in all cells of the body to remove toxic and damaged material. Thus, a decrement to autophagy promotes the accumulation of material that is normally removed from the cell and adversely affects cell survival.

During autophagy, the process of cargo selection is generally non-selective; however, specific autophagy receptors have been identified that mediate the clearance of specific cargoes (Bento et al., 2016). One of the most studied autophagy receptors is the protein p62/SQSTM1, which is both a selective autophagy substrate and a cargo receptor for autophagic degradation of polyubiquitinated proteins (Katsuragi et al., 2015; Bento et al., 2016). p62/SQSTM1 contains a light chain 3 (LC3)-interacting region (LIR), which targets the LC3 anchored in the omegasome membrane; cargoes are thus directed to the nascent autophagosome for clearance (Birgisdottir et al., 2013). Other LIR-containing autophagy receptors are the proteins B-cell lymphoma 2 (Bcl2) Interacting Protein 3 (BNIP3) and NIX, two homologous Bcl2 homology 3 (BH3)-only proteins, which localize in the outer mitochondrial membrane and mediate mitophagy (autophagic clearance of mitochondria) (Kanki, 2010; Hanna et al., 2012). Both BNIP3 and NIX contain a LIR motif in the amino terminus that faces the cytosol, leading to LIR-mediated mitophagy by interaction of the LIR motif with LC3 during autophagosome formation (Kanki, 2010; Hanna et al., 2012).

As mentioned above, the degradation of the autophagic cargo is dependent on lysosomal enzymes. Impaired fusion of the autophagosome with the lysosome, or inhibition of the acidification of the lysosomal compartment prevents the degradation of the autophagosome luminal content and promotes autophagosome accumulation, thus inhibiting “autophagic flux” (Klionsky et al., 2016). The term “autophagic flux” is defined as the difference between the rate of autophagosome formation and cargo clearance by lysosomes (Klionsky et al., 2016). A blockage in the autophagic flux can be observed in different human diseases, and is quite common in neurodegenerative diseases, where an abnormal accumulation of autophagosomes caused by inefficient fusion with lysosomes occurs (Ravikumar et al., 2005). The mechanisms responsible for impairing autophagic flux in neurodegenerative disease vary, and represent an active subject of investigation.

### The autophagy machinery

The site where autophagosome assembly occurs in the cell remains controversial, and different locations and organelles have been proposed as the site of assembly and source of the membranes composing the autophagic vacuoles. The ER (Hayashi-Nishino et al., 2009; Yla-Anttila et al., 2009), mitochondria (Hailey et al., 2010), the plasma membrane (Ravikumar et al., 2010; Moreau and Rubinsztein, 2012), the Golgi apparatus (Geng et al., 2010; Bodemann et al., 2011) and mitochondria-associated membranes (MAMs) (Hamasaki et al., 2013; Patergnani et al., 2015) have all been described as subcellular compartments from which the phagophore and the autophagosome might arise. More than 35 autophagy-related (*atg*) genes are involved in the process of autophagic vacuole formation (Bento et al., 2016). The primitive phagophore becomes an autophagosome thanks to the activity of the class III phosphoinositide 3-kinase (PI3KC3), which is a large macromolecular complex formed by a catalytic sub-unit the vacuolar protein sorting 34 (VPS34), a regulatory sub-unit VPS15 (p150), ATG6 (also known as Beclin 1 in mammals) and ATG14L (Russell et al., 2014). The PI3KC3 also binds, through Beclin 1, the activating molecule in Beclin 1-related autophagy 1 protein (AMBRA1), which stimulates the activation complex when autophagy is induced (Di Bartolomeo et al., 2010). The pro-autophagy role of Beclin 1 in the PI3KC3 can be also regulated by Bcl-2 or Bcl-XL located at the ER, which down-regulates autophagy by binding Beclin 1, specifically through the Bcl-2 homology type 3 (BH3) domain (Maiuri et al., 2007a,b).

The increased activity of PI3KC3 generates phosphatidylinositol 3-phosphate (PtdIns3P) in membrane microdomains where proteins with the PtdIns3P-binding (FYVE) and the conserved Phox-homology (PX) domains are recruited to form a protein elongation platform at the omegasome (Simonsen et al., 2004; Zhong et al., 2009). One of these proteins with FYVE domains is the double FYVE domain-containing protein 1 (DFCP1), which is highly accumulated in a PtdIns3P-dependent manner in conditions of starvation-induced autophagy (Axe et al., 2008). Other proteins whose recruitment is mediated by PtdIns3P are the WD-repeat proteins interacting with phosphoinositides, WIPI-49, which is four member protein family: WIPI1, WIPI2, WIPI3, and WIPI4 (Proikas-Cezanne et al., 2004).

The elongation and expansion of the phagophore requires the concerted actions of two ubiquitin-like conjugation systems (Polson et al., 2010). The first ubiquitin-like conjugation system is formed by ATG12–ATG5–ATG16L. ATG16L1, which is directly bound through WIPI2 to the membrane micro domains, allows the expansion of the phagophore (Itakura and Mizushima, 2010; Walczak and Martens, 2013). Specifically, the activity of this ubiquitin-like conjugation system begins with the conjugation of ATG12 to the lysine residue of ATG5 by the sequential reactions of the E1-like enzyme ATG7, and the E2-like enzyme ATG10. The resulting ATG12–ATG5 conjugate interacts non-covalently with ATG16L, and they oligomeryze, forming a large multimeric complex (Hamasaki et al., 2013). In the second ubiquitin-like system, the cytosolic forms of ATG8 (microtubule-associated protein 1 light chain 3 (MAP-LC3), GABARAP and GATE-16) are cleaved at their C-terminus by the cysteine protease ATG4B and then conjugated at the amino group with the lipid phosphatidylethanolamine (PE) by ATG7 and by the E2-like enzyme ATG3 (Tanida et al., 2004; Katahira et al., 2008). LC3 is the most characterized ATG8 family member and its lipidation with PE generates LC3-II (Otomo et al., 2013). LC3-II is specifically bound to the autophagic vesicle and is widely used as an autophagic marker (Klionsky et al., 2016). Once the autophagosome is formed, the ATG12-ATG5-ATG16L complex associated to the convex surface of the autophagosomal membrane dissociates and is released into the cytoplasm to be reused for the biogenesis of new autophagy vesicles (Lamb et al., 2013).

Once the autophagosomes are fused with the lysosomes, hydrolytic lysosomal enzymes are activated, and they degrade both the luminal content of the autophagosome and its inner membrane. This process of degradation allows the formation of new metabolites that recycle back to the cytoplasm for being reused (Lamb et al., 2013). Autophagosomes fuse with lysosomes by the action of several soluble N-ethylmaleimide-sensitive fusion (NSF) attachment protein receptors (SNARE). These include the vesicle-associated membrane protein (VAMP)8, syntaxin-7, syntaxin-8, and t-SNAREs homolog 1B (Vti1B) (Moreau et al., 2013). Impairment in lysosome-autophagosome fusion induced by dysfunctions in SNARESs proteins decreases the size of the autophagic vesicles and inhibits their maturation into autophagolysosomes. This blockage in the fusion process leads to lysosomal storage disorders, which are characterized by an abnormal accumulation of substrates in the lysosomes (Fraldi et al., 2010; Moreau et al., 2013).

**Figure 2:**
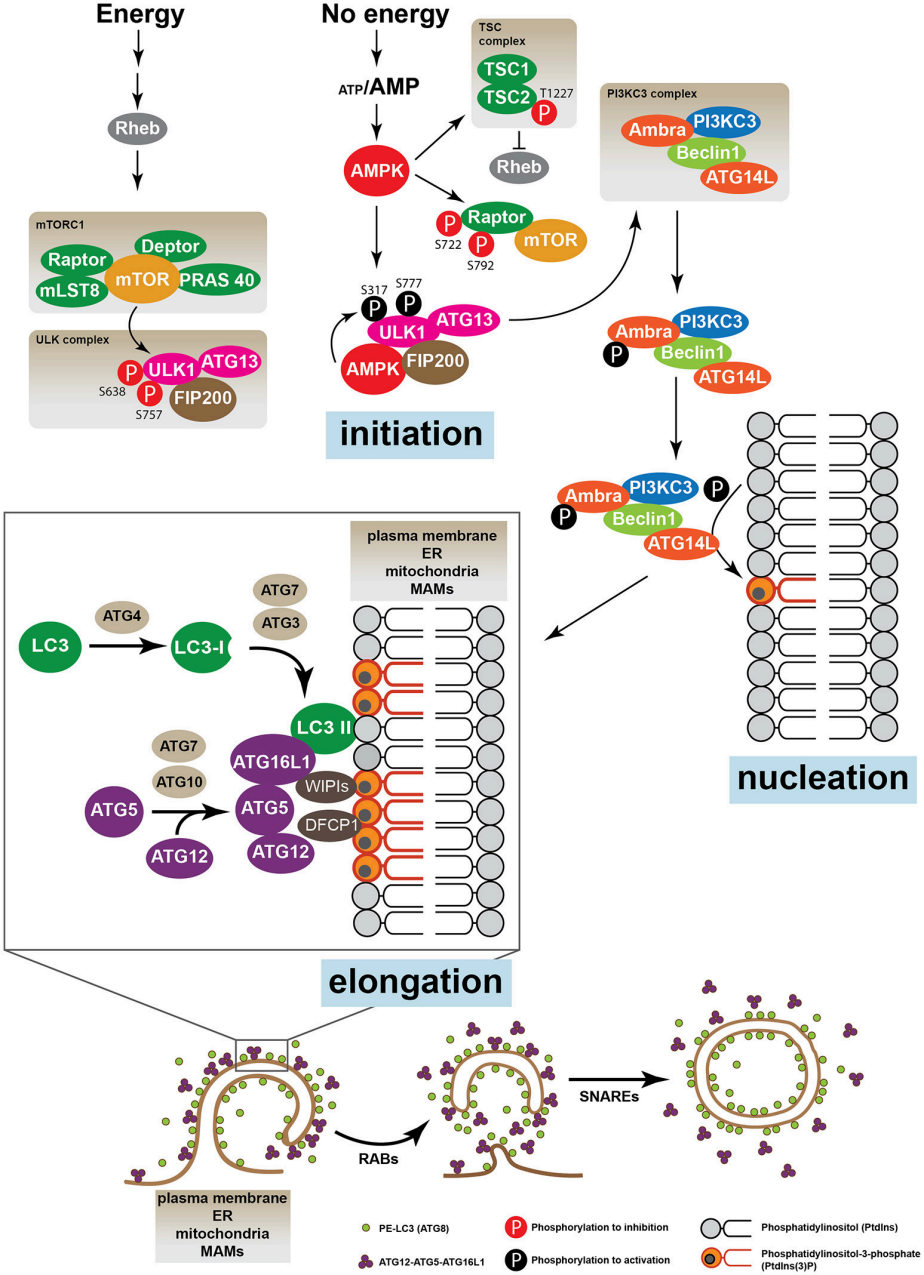
The autophagic machinery. The energetic status of the cell is sensed by the proteins mTOR and AMPK. When nutrients are high mTOR, which, together with Raptor, Deptor, mLST8, and PRAS 40 forms the mTORC1 complex, inhibits the ULK1 kinase activity phosphorylating its Ser638 and Ser757. ULK1, ATG13, and FIP200 form the ULK complex. Upon nutrient deprivation, AMPK is activated and induces autophagy phosphorylating different target proteins. Unlike mTOR, AMPK activates ULK1 by phosphorylation on Ser317 and Ser777, leading to the activation of the PI3KC3 complex, which is formed by AMBRA, PI3KC3, Beclin 1 and ATG14L. AMPK can also induce autophagy by direct suppression of the mTOR pathway phosphorylating Raptor (Ser722 and Ser792) or TSC2 (Thr1227). Active PI3KC3 increases the production of phosphatidylinositol 3-phosphate (PtdIns3P) in specific membrane micro domains of the plasma membrane, the ER, mitochondria and/or MAMs. Accumulation of PtdIns3P recruits FYVE domains proteins such as WIPIs and DFCP1. The elongation and expansion of the phagophore membrane are mediated by ATG proteins through two ubiquitination-like systems: the complex ATG12-ATG5-ATG16L and the conjugate ATG8 (LC3)-phosphatidylethanolamine (PE) (also called LC3-II). Finally, while the ATG12-ATG5-ATG16L conjugate is disassembled from the autophagosomal membrane, LC3-II remains enclosed to the inner and the external membrane of the vacuole. Other proteins such as SNARES and RABs are also required for the trafficking of membranes and vacuole elongation.

In the regulation of the autophagic process, the most characterized protein regulators are the mechanistic target of rapamycin, mTOR, and the AMP-activated protein kinase (AMPK), which are considered cellular nutritional sensors able to modulate different signaling pathways (Kim et al., 2011; Meijer et al., 2015; Chantranupong and Sabatini, 2016). mTOR is a constitutive Ser/Thr protein kinase sensitive to nutritional and energetic changes, and comprises two large protein complexes, the mTOR complex 1 (mTORC1) and mTOR complex 2 (mTORC2) (Russell et al., 2014). mTORC1 is formed by mTOR, Regulatory-associated protein of mTOR (RAPTOR), mTOR associated protein, LST8 Homolog (mLST8), DEP domain containing mTOR-interacting protein (DEPTOR) and proline-rich Akt substrate of 40 kDa (PRAS40). In basal conditions the kinase activity of mTOR renders unc-51-like kinase 1 protein (ULK1, ortholog of Atg1 in humans) inactive by phosphorylation of Ser757, Ser758 and Ser638 (Kim et al., 2011; Meijer et al., 2015). In these conditions, the ULK complex formed by ULK1, ATG13 and FAK family kinase-interacting protein of 200 kDa (FIP200) remains inactive and autophagy is inhibited (Hara et al., 2008; Wong et al., 2013). In conditions of nutrient deprivation, a low ATP/AMP ratio activates AMP-activated protein kinase (AMPK), which up-regulates the Ser/Thr kinase activity of ULK by phosphorylation on Ser317, Ser777, and Ser555 (Wong et al., 2013). Once activated ULK is released from mTORC1 and relocates to phagophore assembly sites where it increases the activity of PI3KC3, which, as previously mentioned, generates PtdIns3P in the membrane microdomains (Axe et al., 2008). AMBRA can also regulate the relocalization of PI3KC3 to the autophagosome nucleation sites (Di Bartolomeo et al., 2010). Once autophagy is induced, ULK1 phosphorylates AMBRA, which releases PI3KC3 from the microtubule-associated dynein motor complex, initiating the process of autophagosome elongation (Di Bartolomeo et al., 2010) (Figure 2).

On the other hand, AMPK up-regulates autophagy through phosphorylation of tuberous sclerosis complex 2 (TSC2) at Thr1227, which, together with TSC1, forms the TSC complex (Inoki et al., 2003; Orlova and Crino, 2010). Active TSC2 induces autophagy by suppression of the RAS homolog enriched in brain (Rheb) protein, which is required to maintain mTOR constitutive activity (Orlova and Crino, 2010). AMPK can also directly phosphorylate the mTORC1 subunit RAPTOR, which inhibits mTORC1, thereby inducing autophagy (Gwinn et al., 2008) (Figure 2).

The protein kinase B (PKB, also known as AKT) is another negative regulator of autophagy (Heras-Sandoval et al., 2014). When nutrients and growth factors are provided, tyrosine kinase receptors are activated by autophosphorylation and stimulate the small GTPase Ras and class I Phosphatidylinositol 3 kinase (PtdIns3K) (Russell et al., 2014). Active class I PtdIns3K increases the levels of PtdIns(3)P in the membrane, promoting the recruitment and the subsequent activation of AKT (Russell et al., 2014). AKT phosphorylates TSC2 on multiple sites, leading to the disruption of the TSC1-TSC2 complex and promoting mTORC1 activation, thus inhibiting autophagosome formation (Inoki et al., 2002; Potter et al., 2002).

## Autophagy dysregulation in neurodegenerative diseases

Autophagy dysfunction has been identified as a characteristic of the vast majority of neurodegenerative diseases. Components of the autophagic machinery, involved in the different stages of autophagosome formation up to autophagosome-lysosome fusion, can be altered in these illnesses, inhibiting autophagy at various stages along the pathway. This generates different pathological situations, generally characterized by the deposition of aggregate prone proteins in neurons.

Consistently, neuron-specific deletion of essential autophagy genes (*Atg5* and *Atg7*) inhibits autophagy and promotes a neurodegenerative phenotype characterized by axonal degeneration and accumulation of aggregate-prone proteins in the cytosol of neurons without altering proteasome function (Hara et al., 2006; Komatsu et al., 2006). These studies indicate that basal autophagy is required to prevent the accumulation of damaged proteins and toxic material, which, if allowed to accumulate, leads to neurodegeneration. Autophagy is particularly relevant in neurons, which are post-mitotic cells. Without the aid of cell division, neurons require efficient autophagy to remove the waste material that would otherwise accumulate in the cytosol. Additionally, neuronal autophagy has been shown to be essential not only for proper neuronal function, but also for prevention of inflammation in glial cells, as well as proper oligodendrocyte development and preservation of the myelination process (Lee, 2012).

As indicated in the previous section, the autophagic process includes multiple steps, each of which can be altered in the different types of neurodegenerative diseases.

### Dysfunctional formation of the isolation membrane

In some mouse models of Alzheimer's disease (AD) the gene encoding for Beclin 1 shows decreased mRNA levels in brain tissue, suggesting reduced autophagosome formation and increased development of AD (Pickford et al., 2008). Consistently, crossing mice that overexpress the human amyloid precursor protein, with those carrying a heterozygous deletion of Beclin 1, which *per se* leads to neurodegeneration, aggravates the AD phenotype (Pickford et al., 2008). Beclin 1 activity is also affected in Huntington disease (HD). Indeed, the mutant Huntington gene, which is critical for the onset of HD, recruits Beclin 1, inhibits its activity and, therefore, autophagosome formation (Shibata et al., 2006). Also, mTOR appears to be sequestered in cell and mouse models of HD, as well as in human brains of HD patients, indicating that the initiation of the autophagic process has been inhibited (Ravikumar et al., 2004).

Additionally, the first step of the autophagic process is compromised in some cases of Parkinson's disease (PD), where omegasomes formation is inhibited due to the mislocalization of the protein ATG9, preventing the formation of new autophagosomes. This reduces the clearance of the protein α-synuclein, which accumulates in intra-neuronal inclusions called Lewy bodies, a feature of the brain tissue of PD patients (Winslow et al., 2010).

### Cargo sequestration

Inefficient recognition of aggregate proteins during the autophagic process has been proposed as a cause of the development of neurodegenerative diseases (Sarkar et al., 2009). As previously described, recognition of certain posttranslational modifications by molecules that bind both cargo and components of the autophagic machinery drives the selectivity of the autophagic cargo (Sarkar et al., 2009). For instance, the protein p62/SQSTM1 is specifically recognized and degraded within autophagosomes (Bjorkoy et al., 2005). Mutations in p62/SQSTM1 have been found in ALS patients, which, as will be better explained in the next sections, leads to p62/SQSTM1 accumulation in inclusion bodies, a typical feature of the disease (Fecto et al., 2011). Also, some forms of HD are characterized by alterations in the process of cargo sequestration. Indeed, due to mutations in the Huntington protein, cellular and mouse models of HD show an inefficient engulfment of cytosolic components in the lumen of autophagosomes, which, even though they form normally, remain empty, thereby promoting the accumulation of the waste material in the cytoplasm of neurons (Martinez-Vicente et al., 2010).

### Autophagosome-lysosome fusion

The next step of the autophagic process is the fusion of autophagosomes with lysosomes or endosomes (to later fuse with lysosomes). Autophagosomes move along microtubules in the direction of the perinuclear microtubule-organizing center of the neuron, which is enriched in lysosomes (Lee et al., 2011). Microtubule-based vesicle transport is necessary for efficient autophagy and, when microtubules are disassembled, autophagosomes accumulate in axonal terminals where they cannot be degraded (Hollenbeck, 1993). Mutations that affect Dynein, a protein that promotes the movement on microtubules, impairs autophagosome-lysosome fusion and thus autophagic clearance of aggregate-prone proteins, causing HD in fly and mouse models (Ravikumar et al., 2005). Similarly, the dynein motor complex is mutated in the axonal Charcot-Marie-Tooth hereditary neuropathy type 2 (CMT2) and loses its function in ALS, promoting autophagosome accumulation (Ferrucci et al., 2011; Sasaki, 2011). Another protein that is necessary for autophagosome-lysosome fusion is RAB7, a late endosome protein, which influences lysosome positioning and thus the autophagosome-lysosome fusion process (Dodson et al., 2012; Uusi-Rauva et al., 2012). This protein has been found mutated in some forms of CMT2 (Spinosa et al., 2008). Additionally, mutations in the protein charged multivesicular body protein 2B (CHMP2B) affecting the activity of the endosomal sorting complex required for transport (ESCRT) have been identified in ALS and FTLD which prevent the formation of the amphisomes, the vesicles that arise from the fusion of the autophagosome with the endosome and ultimately fuse with the lysosome.

Importantly, the many of the details of the autophagosome-lysosome fusion process remain unknown. It is likely that additional proteins are involved in this step of autophagy and their mutation/deletion would likely affect autophagosome turnover.

### Autophagosomal clearance

Once autophagosomes have fused with lysosomes the process of degradation of the cytosolic cargo occurs. Various neurodegenerative diseases are characterized by reduced autophagic flux (increased numbers of autophagosomes). Inefficient autophagosomal clearance can be associated with reduced lysosomal acidification, which reduces the activity of lysosomal hydrolases and promotes the accumulation of undigested byproducts. This is typical of the so-called “lysosomal storage disorders” (LSD), which comprise more than 50 diseases and represent the most common forms of neurodegenerative disease in childhood. While the proteins and mechanisms involved in these diseases differ, impaired autophagy is unfailingly present (Menzies et al., 2015). For example, some forms of PD are characterized by lysosomal dysfunction, which is caused by loss of function mutations in the gene coding for the glucocerebrosidase. This promotes the accumulation of glucocerebroside together with α-synuclein in the brain, thus promoting neurodegeneration (Mazzulli et al., 2011; Murphy et al., 2014). In AD, however, decreased autophagosomal clearance is associated with mutations in the protein presenilin 1, which increases lysosomal pH, reduces lysosomal activity, thereby promoting amyloid deposition and the onset of the disease (Cataldo et al., 2004).

## TDP-43 and C9orf72: roles in neurodegenerative diseases

### The TDP-43 protein

**Figure 3:**
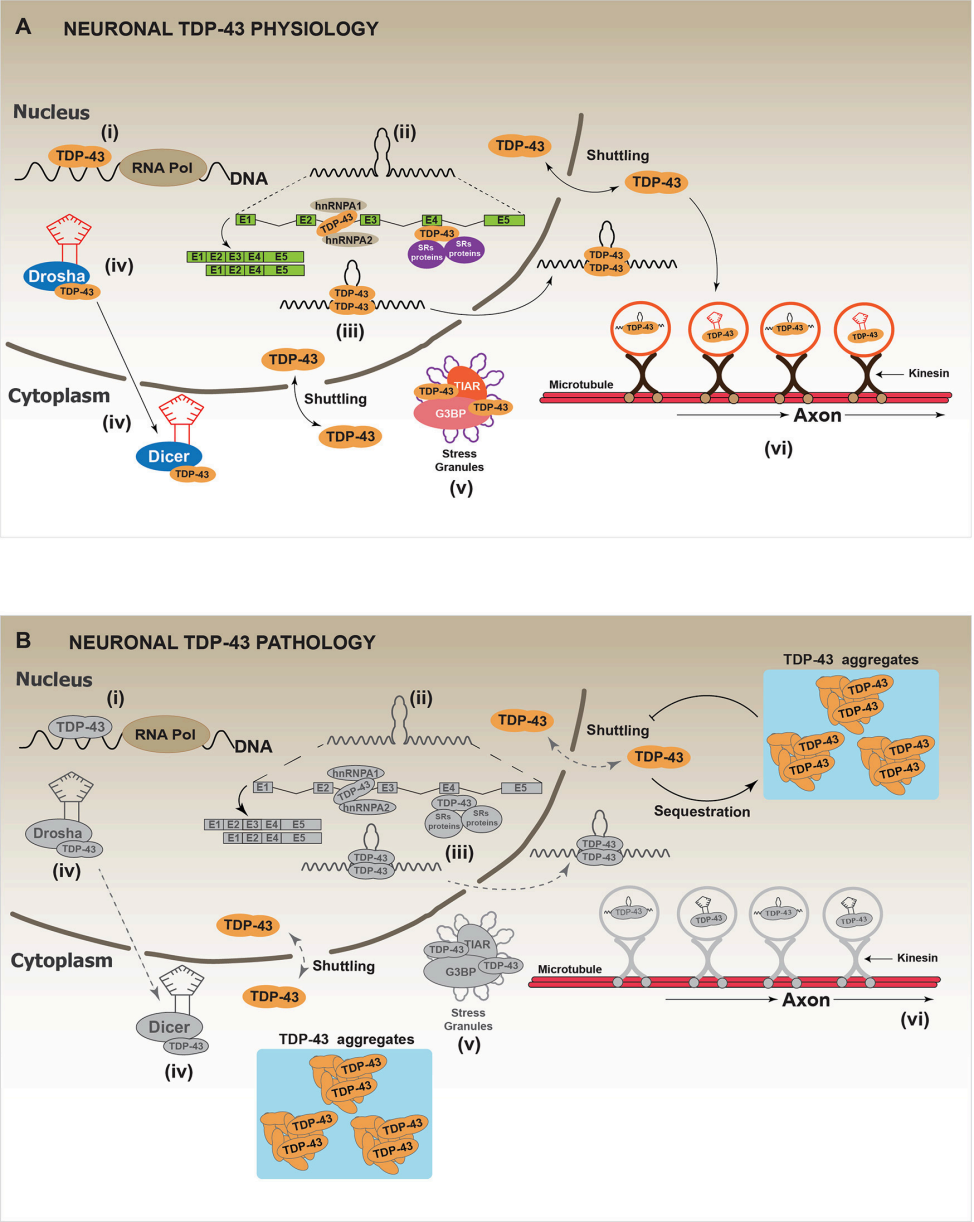
Physiology and pathology of TDP-43. **(A)** In physiological conditions, thanks to its capacity to shuttle between the nucleus and the cytoplasm, TDP-43 regulates several processes that impact on the cellular fate of different RNAs. These processes are: (i) RNA transcription: it includes the regulation of RNA transcription through the direct binding of TDP-43 to promoter regions. (ii) mRNA alternative splicing: the most studied and known function of TDP-43 is the regulation of alternative mRNA splicing. TDP-43 binds to exon or intron regions and recruits other RNA binding proteins (RBPs) such as hnRNPs or SRs proteins (heterogeneous nuclear ribonucleoproteins and serine-arginine proteins, respectively). (iii) RNA stabilization: RNA stabilization has been proposed as a function of TDP-43. TDP-43, binding to different mRNAs or miRNAs inhibits their premature degradation and possibly favors their translocation from the nucleus to the cytoplasm. Different studies report a decrease in the half-life of several mRNAs and miRNAs after TDP-43 down-regulation. (iv) miRNAs synthesis and processing: TDP-43 has been implicated in the synthesis and processing of different miRNAs by interacting with Drosha and Dicer complexes. It has been reported that TDP-43 down-regulation affects the cellular levels of several miRNAs. (v) Stress granules localization/regulation: In different conditions of cellular stress, it has been shown that TDP-43 is located in the stress granules (SGs) where it interacts with classical SGs proteins such as TIAR and G3BP. Although the real function of TDP-43 in SGs is still unknown, it has been reported that TDP-43 down-regulation affects SGs formation by decreasing G3BP mRNA levels. (vi) RNA transport: Data indicate TDP-43 is required for the transport of different RNAs along the neuronal axon, by a mechanism that might involve microtubules and kinesin complexes. **(B)** In a pathological situation, TDP-43 forms cellular aggregates, mainly in the cell cytoplasm, through a mechanism that is currently unknown. It has been proposed that TDP-43 aggregates can be toxic for the cell. However, a generally accepted hypothesis suggests that TDP-43 aggregates could act as “sink” that continuously sequesters functional TDP-43 protein, thus, increasing the aggregation process by a positive feed-back loop. This would lead to a generalized TDP-43 loss-of-function, which promotes cellular stress and eventually leads to cell death. Gray color: represents attenuated processes due to TDP-43 loss-of-function.

TAR DNA-binding protein (TARDBP), hereafter called TDP-43, was initially described as a transcriptional repressor factor of HIV (Ou et al., 1995). Since then, numerous studies have established that TDP-43 is a RNA binding protein that participates in multiple pathways of the “RNA metabolism,” which includes RNA splicing, RNA transport, mRNA stability, microRNA processing and stress granules assembly (Aulas et al., 2012; Buratti and Baralle, 2012; Ratti and Buratti, 2016) (Figure 3A). From a molecular point of view, the N-terminal of TDP-43 contains a nuclear localization signal (NLS), a nuclear exclusion signal (NES) and two RNA binding domains (RMM1, RMM2). The presence of NLS and NES allows the shuttling of TDP-43 between the nucleus and the cytoplasm (Ayala et al., 2008; Prpar Mihevc et al., 2016). The RRM1 is the region through which TDP-43 binds different RNAs targets (Buratti and Baralle, 2001; Ayala et al., 2011; Avendaño-Vázquez et al., 2012). Additional studies have suggested that RRM2 is required to bind the DNA and to maintain the protein structure (Kuo et al., 2009). Finally, the C-terminal region of TDP-43 is composed by a glycine rich domain (GRD), which has been involved in most of protein-protein interactions (Buratti et al., 2005; D'ambrogio et al., 2009; Buratti and Baralle, 2012). The GRD domain includes a glutamine/asparagine (Q/N) prion-like domain responsible for both, protein-protein interactions (Budini et al., 2012a) and TDP-43 aggregation (Fuentealba et al., 2010; Budini et al., 2012a,b). In addition to the Q/N region, also the N-terminal region of TDP-43 has been implicated in the aggregation process. Indeed, this domain is required to sequester the native TDP-43 into the aggregates, causing a loss-of-function (Figure 3B) (Budini et al., 2015; De Conti et al., 2015; Romano et al., 2015; Langellotti et al., 2016).

TDP-43 is the major component of inclusions or aggregates present in neuronal cells from patients affected by ALS and FTLD (Arai et al., 2006; Neumann et al., 2006). Studies have shown that more than forty disease-associated mutations have been discovered in TDP-43, principally in the C-terminal region of the protein (Buratti and Baralle, 2009; Mackenzie et al., 2010; Lattante et al., 2013). Even though experimental data suggest that some TDP-43 mutations favor its aggregation (Budini et al., 2012b), it is worth to note that around the 95% of people showing TDP-43 inclusions the protein is not mutated (Lattante et al., 2015). Hence, TDP-43 aggregation process does not necessarily require protein mutations.

As previously mentioned, TDP-43 shuttles between the nucleus and the cytoplasm (Ayala et al., 2008), however, in pathological conditions, it has been observed that TDP-43 mainly remains in the cytoplasm where it can aggregate. Indeed, affected cells can show cytoplasmic TDP-43 aggregates and complete depletion of TDP-43 in the nuclear compartment (Arai et al., 2006; Neumann et al., 2006). TDP-43 aggregates are mainly composed by TDP-43 full-length and by C-terminal protein fragments (25 and 35 kDa) that can be distinguished by ubiquitination and specific phosphorylation (Arai et al., 2006; Neumann et al., 2006; Hasegawa et al., 2008; Inukai et al., 2008).

On the other hand, mitochondrial dysfunction, observed in samples from patients with ALS, may be regulated by TDP-43. Indeed, overexpression of TDP-43 and its C-terminal fragments induced mitochondrial damage in NSC-34 motor neuron cell line (Hong et al., 2012). In addition, full-length TDP-43 and truncated TDP-43 localize in the mitochondria, suggesting that TDP-43 and its C-terminal fragments cause mitochondrial dysfunction and enhance mitophagy (Hong et al., 2012). These observations were also confirmed by Onesto et al. (2016), who reported that the overexpression of a pathological A382T TDP-43 mutant in fibroblasts, induced changes in mitochondria morphology which caused the fragmentation of the mitochondria network (Onesto et al., 2016). Thus, since mitochondria are key in neuronal metabolism, TDP-43 mutations can induce cell death by impairing not only TDP-43-dependent RNA metabolism, but also mitochondria function in ALS. In line with this, it was demonstrated that lithium prevents most ALS pathological changes *in vitro* an *in vivo* models by mechanisms that involve mitochondrial protection, induction of autophagy, mitophagy and mitochondriogenesis (Sarkar et al., 2005; Tasdemir et al., 2007; Pasquali et al., 2009; Natale et al., 2015).

Currently, the role of TDP-43 aggregates in neurodegenerative diseases is unknown and two hypotheses have been proposed (Lee et al., 2012): (1) the “gain-of-function” hypothesis, which suggests that cytoplasmic TDP-43 aggregates, or the presence of missense mutations in TDP-43, provokes cell death by cytotoxic effects (Johnson et al., 2008, 2009; Zhang et al., 2009); (2) the “loss-of-function” hypothesis, which suggests that TDP-43 aggregates act as a “sink” by additionally sequestering functional TDP-43 (nuclear and cytoplasmic). This causes a generalized impairment in TDP-43 activity that affects the whole cell physiology (Figure 3B). Despite studies strongly support the idea that the aggregation induces TDP-43 loss-of-function (Budini et al., 2015; De Conti et al., 2015; Romano et al., 2015), it is still not clear which hypothesis is correct and, it is highly possible that a combination of both mechanisms, gain- and loss-of-function, contributes to the onset and disease progression.

### The C9orf72 genetic factor

In addition to TDP-43, other genetic markers have been associated with the onset of ALS and FTLD and, among those, the most studied is the genetic factor C9orf72 (Lattante et al., 2015). This factor, was described in 2011 by two independent groups and consists of a polymorphism composed by different repeats of the hexanucleotide GGGGCC [(G_4_C_2_)n]. These repeats locate in a non-coding region of C9orf72, specifically within the intron that separates exons 1a and 1b (DeJesus-Hernandez et al., 2011; Renton et al., 2011). It has been shown that healthy people carry 20 or less G_4_C_2_ repeats (*n* ≤ 20), whereas affected individuals incorporate between 30 and 250 hexanucleotide repeats (*n* ≥ 30) (Renton et al., 2011). The *C9orf72* gene can generate three different variants by alternative splicing of its pre-mRNA (DeJesus-Hernandez et al., 2011). By analyzing brain samples from patients, studies indicated that the pathological mechanism of (G_4_C_2_)n repeats may involve both, a reduction in the expression of some C9orf72 variants and/or the aberrant formation of RNA foci (DeJesus-Hernandez et al., 2011). Additionally, studies performed during the last 5 years indicated that RNA foci are formed by stable multimeric G-quadruplexes, which can abnormally sequester RNA-binding proteins (RBPs) impairing their physiological functions. In addition, RNA foci can also suffer repeat-associated non-ATG (RAN) translation. This translation occurs in sense and antisense of the (G_4_C_2_)n repeats, in all reading frames, and in the absence of an initiating ATG codon. Thus, this mechanism can generate polypeptides formed by different numbers of dipeptide units originated from one (G_4_C_2_)n repeat. These polypetides, known as DRPs (Dipeptide Repeat Proteins) or C9RANT proteins, have shown to be toxic for the cell, since they impair RNA biogenesis (Zu et al., 2013; Kwon et al., 2014; Gendron et al., 2015). Studies have evaluated the effects of proline/arginine repeated 20 times (PR20) RAN translation products in primary neuronal cultures of rat spinal cord. It has been shown that PR20 induces cell death in motor neurons without causing mitochondrial alterations, ER stress or mTOR inhibition (Gupta et al., 2017). Additionally, PR20 accumulation inhibits protein degradation pathways, such as autophagy and the ubiquitin proteasome system (Gupta et al., 2017). The pathological effects of (G_4_C_2_)n repeats have been recently reviewed by Todd and Petrucelli (2016).

The physiological role of C9orf72 has been poorly studied. Two different groups found that C9orf72 shares structural similarities with proteins containing DENN modules (Zhang et al., 2012; Levine et al., 2013). Proteins containing DENN modules work as GDP-GTP exchanging factors for RAB GTPases, which are involved in endosomal traffic and in the formation of autophagic vacuoles (Zerial and McBride, 2001; Amaya et al., 2015). Thus, it was proposed that C9orf72 has a role in both endosomal traffic and autophagy. Additional studies showed that C9orf72 interacts with different RAB proteins (Farg et al., 2014) involved in the autophagic process, confirming the hypothesis that C9orf72 plays an important role in the autophagic pathway (Amaya et al., 2015).

## Role of autophagy in TDP-43 and C9orf72 related diseases

### Impaired autophagy in ALS and FTLD

Studies have demonstrated that mutations in autophagy-related genes are associated with familial ALS and FTLD (Skibinski et al., 2005; Parkinson et al., 2006; Lee et al., 2009; Maruyama et al., 2010; Le Ber et al., 2013; Shimizu et al., 2013; Teyssou et al., 2013; Wong and Holzbaur, 2014; Cirulli et al., 2015; Krasniak and Ahmad, 2016). In fact, mutations in the ubiquitin-binding site on *p62/SQSTM1* have been associated with ALS and FTLD caused by the inhibition of protein aggregates clearance mediated by autophagy (Le Ber et al., 2013; Shimizu et al., 2013; Teyssou et al., 2013). Other genes, whose mutations are associated with impaired autophagy regulation, are *Optineurin (optn)* and *CHMP2B*. OPTN, which is involved in the mitophagy machinery, is proposed to impact on ALS as its mutations promote severe mitochondria degradation (Maruyama et al., 2010; Wong and Holzbaur, 2014). CHMP2B, which is part of the ESCRTIII protein complex, is involved both in the endosomal-sorting pathway (Skibinski et al., 2005; Parkinson et al., 2006) and in the formation of autophagolysosomes (Filimonenko et al., 2007). In agreement with this, it has been observed that mutations in *CHMP2B* promote autophagic vesicles accumulation in brain tissues of FTLD patients (Lee et al., 2009; Krasniak and Ahmad, 2016).

Furthermore, new genetic factors that associate the autophagic machinery and neurodegenerative diseases have been identified. A recent exome sequencing assay performed on more than 2,000 ALS patients identified the *TANK-Binding Kinase 1* (*Tbk1*) as a new gene associated with ALS (Cirulli et al., 2015). TBK1 protein belongs to the IKK-kinase family, which is involved in immune signaling pathways in neurons and other cell types (Oakes et al., 2017). In this regard, studies have suggested that TBK1 may be implicated in protein aggregations-induced neuroinflammation (Oakes et al., 2017). Other studies have shown that TBK1 phosphorylates OPTN and p62/SQSTM1 to promote autophagy, and, when non-functional, TBK1 can impair autophagy and mitophagy, affecting neuronal homeostasis (Matsumoto et al., 2015; Richter et al., 2016).

Altogether, these studies suggest that mutations in autophagy-related genes and, as consequence, impaired autophagy in the brain tissue might contribute to the onset or progression of ALS and FTLD.

### Autophagy AND TDP-43

#### Regulation of TDP-43 by autophagy

Studies have shown that autophagy vacuole proteins such as LC3 and p62/SQSTM1 co-localize with TPD-43 aggregates, supporting the hypothesis that physiological rates of autophagy are required to avoid the accumulation of TDP-43 aggregates (Mizuno et al., 2006; Hiji et al., 2008). Indeed, it was demonstrated, using different approaches, that TDP-43 inclusions on spinal cord samples from ALS and FTLD patients are also positive for p62/SQSTM1 (Mizuno et al., 2006; Hiji et al., 2008). In addition, other studies have suggested that the co-localization between TDP-43, p62/SQSTM1 and ubiquitin is common in sporadic and familial ALS cases as well as in other TDP-43 proteinopathies (Maekawa et al., 2009; King et al., 2011).

In addition to the autophagic vacuole markers p62/SQSTM1 and LC3, also autophagy regulator proteins have been identified co-localizing with TDP-43 inclusions. This is the case of Vasolin-containing protein (VCP) and OPTN, which co-localize with TDP-43, p62/SQSTM1 and ubiquitin in spinal motor neurons of sporadic ALS patients (Ayaki et al., 2014). Thus, the identification of vacuole autophagy markers or autophagy regulator proteins in TDP-43 inclusions suggests that autophagy can be up-regulated to prevent the pathological accumulation of TDP-43 aggregates.

Given that protein inclusions co-localize with autophagic vacuole markers in samples from patients affected by TDP-43-dependent proteinopathies, different groups have evaluated whether autophagy regulates the formation of TDP-43 aggregates. Caccamo et al. (2009) showed that inhibition of autophagy by treatment with 3-Methyladenine (3-MA), promotes the accumulation of full-length TDP-43 and its 35 and 25 kDa degradation products in N2a and SH-SY5Y cells. Similar results were observed in HEK-293 cells overexpressing GFP-TDP-43 WT or the GFP-TDP-25 kDa fragment, since the treatment with 3-MA increased the levels of the exogenous forms of TDP-43. Consistently, when autophagy was induced in N2a and SH-SY5Y cells by treatment with rapamycin, the degradation of the different forms of TDP-43 was enhanced (Caccamo et al., 2009).

Elevated expression of LC3 has been identified in skin biopsies of ALS patients carrying the TDP-43 A315T mutation, suggesting that this pathogenic TDP-43 mutation might induce autophagy (Wang et al., 2015). In line with this study, Barmada et al. screened over a million of compounds and found that pluphenazine-, methontrimeprazine- and compound 10-(4′-(N-diethylamino)butyl)-2- chlorophenoxazine are able to induce autophagy, enhance the turnover of TDP-43 WT and TDP-43 A315T, and increase the survival of neurons in ALS models (Barmada et al., 2014).

The control of TDP-43 protein levels by autophagy can also be co-regulated by heat shock proteins (HSP). Indeed, it has been observed that HSP-90, cell division cycle 37 (CDC37) and TDP-43 form a protein complex that prevents TDP-43 degradation in HeLa cells. The disruption of this complex, by downregulation of HSP-90 or CDC37 promoted degradation of TDP-43 via induction of autophagy (Jinwal et al., 2012). The same results were found in the neuronal-like cell lines NSC-43 and SH-SY5Y (Crippa et al., 2010a,b, 2016), in which overexpression of the small heat shock protein B8 (HspB8) induced the degradation of TDP-43 WT and its truncated forms, TDP-43 ΔC and TDP-25 kDa by increasing autophagy (Crippa et al., 2010a). Notably, colchicine- and doxorubicin-induced up-regulation of HspB8, enhanced the expression of *tfeb, p62/sqstm1* and *lc3*, suggesting that HspB8 might have a pro-autophagic role, which can be associated to TDP-43 catabolism (Crippa et al., 2016).

As previously mentioned, another adaptor protein involved in TDP-43 degradation through autophagy is OPTN. As indicated, mutations in the *optn* gene have been found in ALS patients (Shen et al., 2015). Consistently, in N2a cells, mutations affecting the ubiquitin binding site of OPTN enhance its accumulation in inclusion bodies (IBs), where TDP-43 has also been identified. These mutations also modify the degradation rate of TDP-43 through autophagy, probably because mutant proteins are unable to interact with myosin VI (MYO6) and with the target of Myb1 membrane trafficking protein (TOM1), thus avoiding the process of autophagosomes maturation (Shen et al., 2015).

Additionally, studies have demonstrated that autophagy and the ubiquitin proteasome system (UPS) are strictly coordinated to degrade WT and aggregated forms of TDP-43 (Urushitani et al., 2010; Wang et al., 2010; Huang et al., 2014). In accordance with this, studies in HEK-293 and SH-SY5Y cells showed that even though WT TDP-43 is mainly degraded by the UPS system, aggregated forms of TDP-43 are preferentially degraded by autophagy (Scotter et al., 2014). Interestingly, the inhibition of both UPS and autophagy induces the aggregation of ΔNLS-TDP-43, a mutant form of TDP-43 unable to localize at the cell nucleus. These data suggest that a combination between an excessive accumulation of cytoplasmic TDP-43 and the impairment in cellular degradative pathways leads to TDP-43 aggregation. Thus, given that more than one protein turnover pathway is involved in the catabolism of TDP-43, it seems that the recycling of TDP-43 must be tightly controlled (Ayala et al., 2011; Avendaño-Vázquez et al., 2012).

*In vivo*, the role of autophagy in the regulation of TDP-43 levels has been poorly analyzed. Studies showed that rapamycin induced-autophagy improved learning and memory in transgenic mice overexpressing WT TDP-43. Treatment with rapamycin, which inhibits mTOR activity, increased LC3 I to LC3 II conversion and the turnover of different forms of TDP-43 (full length, 25 and 35 kDa) (Wang et al., 2012). These studies further support data showing that autophagy is involved in the turnover of full length and aggregated forms of TDP-43.

Altogether, these studies indicate that autophagy is implicated in the control of TDP-43 protein levels, affecting both WT and the aggregated forms of TDP-43 *in vitro* and *in vivo*. Thus, autophagy is crucial in the onset and progression of TDP-43-related neurodegenerative diseases. The discovery of new drugs able to modulate autophagy needs to be considered as a strategy for the treatment of these diseases associated with dysregulated proteostasis of TDP-43.

#### Regulation of autophagy by TDP-43

As previously mentioned, TDP-43 is implicated in the regulation of autophagy. Indeed, mRNA hybridization assays in N2a cells showed that TDP-43 binds to *atg7* mRNA through its RRM1 domain and that down-regulation of TDP-43 decreased *atg7* mRNA levels (Bose et al., 2011). Consistently, cells lacking TDP-43 showed reduced ATG7 protein levels compared to wild-type cells, as well as impairment in basal and stimulated autophagy, as indicated by a reduction in LC3-II and accumulation of p62/SQSTM1, autophagosomes and ubiquitinated inclusions (Bose et al., 2011).

**Figure 4:**
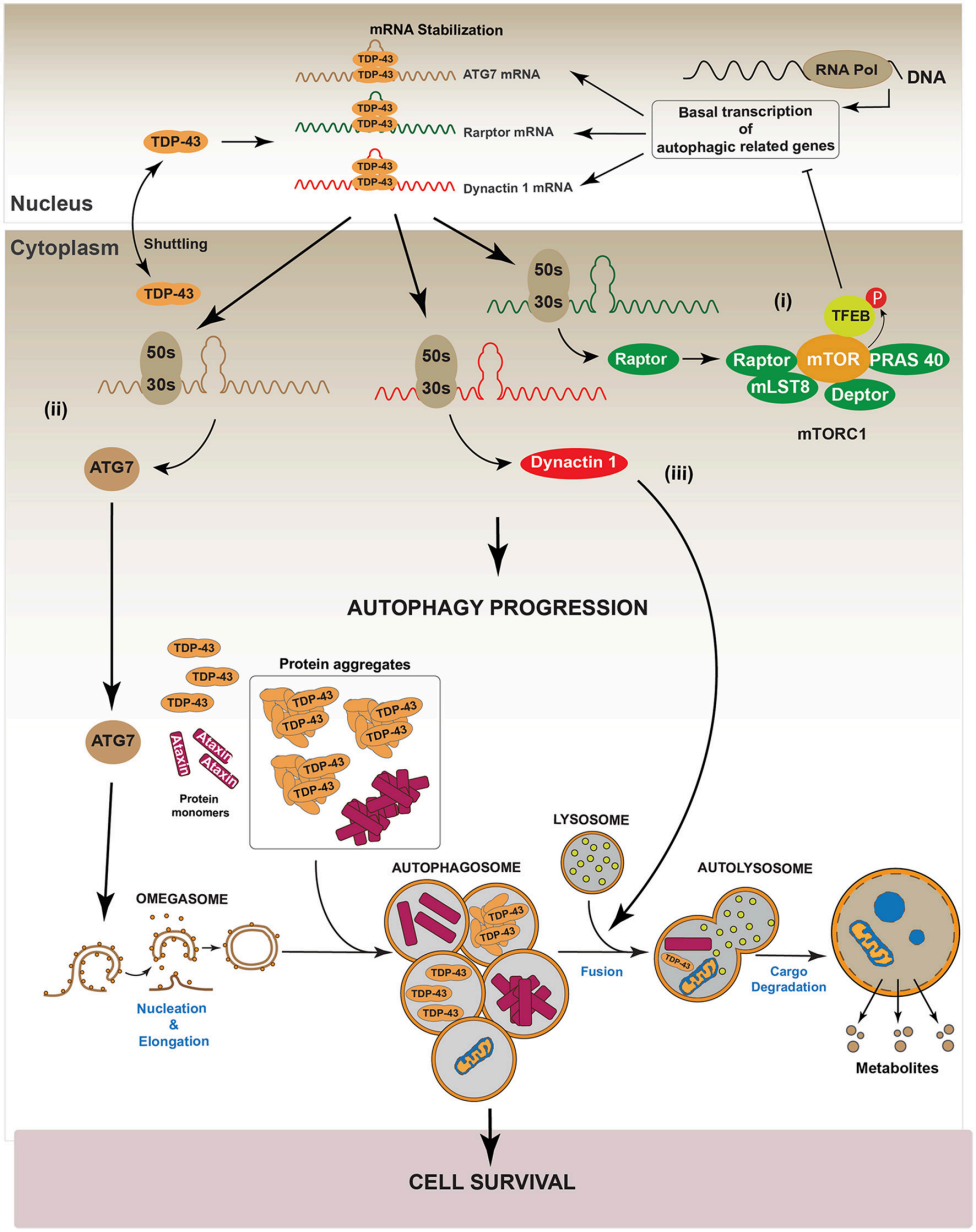
Regulation of autophagy by TDP-43. It has been reported that TDP-43 regulates basal autophagy by stabilizing three different mRNAs: *atg7, raptor*, and *dynactin 1*. The cellular levels of these autophagic mRNAs decrease after TDP-43 down-regulation. Thus, data indicate TDP-43 can participate in autophagy regulation at two different steps: autophagic initiation and autophagic flux progression. (I) Autophagy initiation: TDP-43 stabilizes the mRNA that codes for the protein Raptor, thus ensuring sufficient levels of this protein and keeping mTORC1 active (i). In turn, mTORC1 allows the phosphorylation of TFEB decreasing its nuclear translocation. Thus, by stabilizing mRNA *raptor* levels, TDP-43 ensures the maintenance of basal autophagy. (ii) TDP-43 stabilizes *atg7* mRNA and thus its protein levels. In this way, TDP-43 regulates the initiation step and the formation of new autophagosomes (ii). (II) Autophagic flux progression: by stabilizing *dynactin 1* mRNA levels and Dynactin 1 protein levels, TDP-43 allows the progression of the autophagic flux (iii). The latter, is supported by data showing that Dynactin 1 is required in the process of autophagosome-lysosome fusion.

Recently, Xia et al. showed that TDP-43 down-regulation in HeLa, HEK293, HT22, PC12, and SH-SY5Y cell lines, promotes the nuclear translocation of Transcription Factor EB (TFEB), a master transcription factor that regulates the expression of genes controlling lysosomes biogenesis and autophagy induction (Napolitano and Ballabio, 2016; Xia et al., 2016). In this study authors also reported that TDP-43 controls the level of RAPTOR, which is required to maintain the activity of mTOR, by stabilizing its mRNA (Xia et al., 2016). Thus, silencing of TDP-43 inactivated mTOR by decreasing RAPTOR levels (Xia et al., 2016). Given that phosphorylation of TFEB by mTOR is required to maintain the cytoplasmic location of TFEB, down-regulation of TDP-43 decreased mTOR-dependent TFEB phosphorylation and induced the translocation of TFEB into the nucleus. The augmented nuclear localization of TFEB increased the gene expression of *lamp1, lamp2, atg5, beclin-1, cathepsinL* among others autophagy related genes (Xia et al., 2016). In accordance with this, LC3-II, lysosomes and autophagosomes were also accumulated (Xia et al., 2016). Despite these data, this study also found that down-regulation of TDP-43 induced accumulation of p62/SQSTM1, suggesting that, TDP-43 is also required for an optimal autophagosome-lysosome fusion (Xia et al., 2016). A possible explanation for these observations is that TDP-43 is also necessary to stabilize the mRNA of *Dynactin 1*, which participates in autophagosome-lysosome fusion (Xia et al., 2016). Thus, TDP-43 down-regulation decreases Dynactin 1 protein levels and, as consequence, it impairs autophagosome-lysosome fusion (Xia et al., 2016). In summary, these data indicate that, down-regulation of TDP-43 promotes autophagy through the transcriptional role of nuclear TFEB. However, because TDP-43 is also required for autophagosome-lysosome fusion by regulating Dynactin 1, the lack of TDP-43 finally causes an impairment in the progression of the autophagic flux (Xia et al., 2016). Figure 4 summarizes the role of TDP-43 in the regulation of the autophagic process.

**Figure 5:**
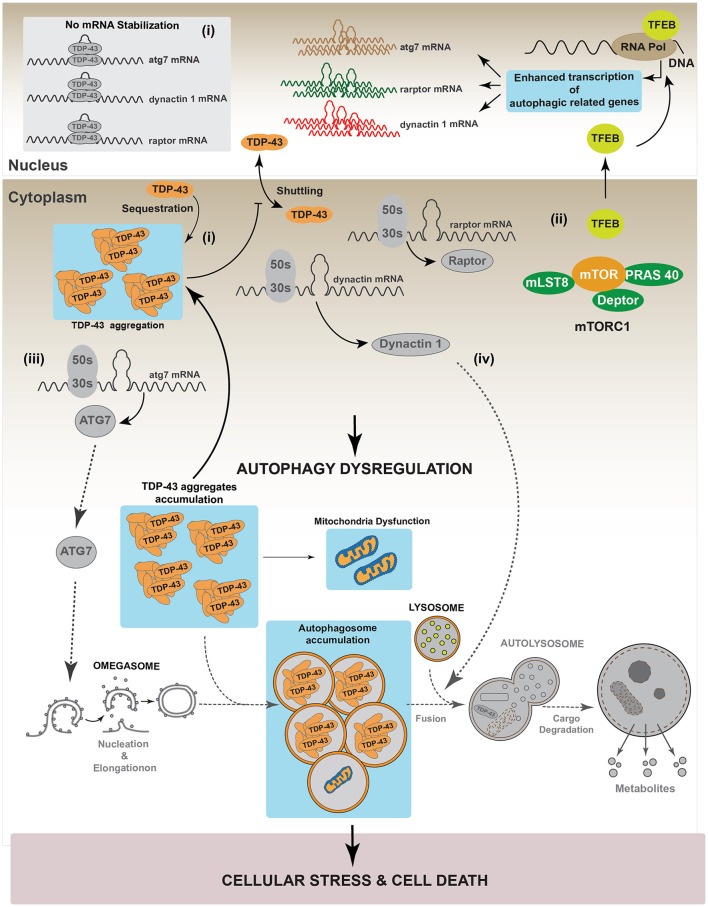
TDP-43 aggregation dysregulates autophagy. In a pathological condition, TDP-43 aggregates might sequester functional TDP-43 causing a complete loss-of-function of this protein. Loss of TDP-43 activity decreases the levels of *raptor, atg7 and dynactin* 1 mRNAs (i). This situation can affect the autophagic pathway in two different ways. (I) Autophagy induction: loss of Raptor protein destabilizes the mTOR complex decreasing the phosphorylation that inhibits TFEB nuclear translocation. Dephosphorylated TFEB in now able to translocate into the nucleus, where it increases the transcription of genes that stimulate autophagy (ii). Loss of TDP-43 activity negatively impacts on the Atg7 protein level, thus decreasing autophagosomes formation (iii). (II) Autophagy progression: this step can be reduced following TDP-43 aggregation as Dynactin 1 protein levels are lower due to TDP-43 loss-of-function (iv). Overall, although the loss of TDP-43 activity increases the expression of genes related with autophagy progression, autophagy is blocked due to the failure in both, autophagosomes formation and autophagosome-lysosome fusion. This situation can promote cellular stress and cell death. Gray color: represents down-regulated proteins and attenuated processes due to TDP-43 loss-of-function.

As mentioned before, studies strongly suggest that TDP-43 aggregates conduce to a TDP-43 loss-of-function (Budini et al., 2015; De Conti et al., 2015). In this condition, the lack of TDP-43 activity may increase cellular stress by inhibiting autophagy. Considering this scenario, the lack of autophagy will further enhance the accumulation of TDP-43 aggregates, leading to an increase in cellular stress, cell death and neurodegeneration (Figure 5). These results are supported by data obtained using a transgenic mouse model which overexpresses the 25 kDa TDP-43 fragment in the brain and in the spinal cord. This transgenic mouse showed reduced autophagy and cognitive alterations as suggested by a decrease in different autophagic markers, such as ATG3, ATG7, LC3-II, p62/SQSTM1 and Beclin 1 (Caccamo et al., 2015).

### Autophagy and C9orf72

#### Regulation of autophagy by C9orf72

The physiological functions of C9orf72 protein have been poorly studied. However, studies published during the last years suggest that C9orf72 plays an important role in the regulation of autophagy, thus impacting the neurodegeneration process. Indeed, ALS and FTLD patients carrying C9orf72 repeats show altered autophagy. Immunohistochemical analysis of 36 patients affected by ALS and FTDL showed a significant accumulation of p62/SQSTM1 in neuronal cells (Al-Sarraj et al., 2011). In addition, tissues from cerebral cortex, basal ganglia, hippocampus and cerebellum from ALS patients showed a strong reactivity for p62/SQSTM1 in association with C9orf72 foci. However, the evaluated samples were negative for TDP-43 inclusions (Cooper-Knock et al., 2012; Troakes et al., 2012). The same results were observed in FTLD patients, in which the hippocampus, cerebellum, frontal and temporal cortex tissues showed accumulated p62/SQSTM1 and C9orf72 foci (Fratta et al., 2013). Furthermore, co-localization between C9orf72 and p62/SQSTM1 was also observed in pluripotent stem cell-derived neurons from patients having a pathological number of G_4_C_2_repeats. Interestingly, reduced cell viability was also observed in these cells (Almeida et al., 2013). Altogether, these studies indicated that the pathological number of G_4_C_2_ repeats in C9orf72 is associated with impaired autophagic flux.

In addition to the clinical observations, bioinformatic studies showed that C9orf72 protein contains a DENN module (Zhang et al., 2012), which works as a GDP-GTP exchange factor for RAB-GTPases involved in membrane trafficking and autophagy (Levine et al., 2013). Consistently, experiments performed on ALS patient samples, neuronal cell lines (N2a, SH-SY5Y) and primary neuronal cell cultures indicated that the C9orf72 protein co-localizes and interacts with RAB1, RAB5, RAB7, and RAB11, which are involved in autophagy (Farg et al., 2014). Furthermore, C9orf72 also locates in autophagosomes and lysosomes, as demonstrated in cells transfected with LC3-DsRed or dyed with Lysotracker. These results indicate that C9orf72 directly affects the autophagic machinery (Farg et al., 2014). Indeed, studies performed in HEK-293, HeLa, SH-SY5Y, primary cortical neurons and induced neuronal progenitor cells (iNPC) have shown that small and large variants of C9orf72 interact with the ULK complex, inducing activation and translocation of ULK to the phagophore assembly site, thus up-regulating autophagy (Webster et al., 2016).

As mentioned before, the interaction of C9orf72 with specific RABs or with the ULK complex positively regulates autophagy (Farg et al., 2014). However, recent studies have shown that C9orf72 has to bind additional key proteins to promote autophagy. In this context, three independent works demonstrated, by affinity purification and mass spectrometry analyses in different cell lines (N2a and HEK-293) that the large variant of C9orf72 interacts with WDR41 and SMCR8 (Sellier et al., 2016; Sullivan et al., 2016; Ying et al., 2016). WDR41 and SMCR8 are two DENN proteins identified as part of the human autophagic interactome (Behrends et al., 2010). These studies showed that the C9orf72/SMCR8/WDR41 complex is required for the recruitment of the autophagic initiation complex FIP200/ULK-1/ATG13/ATG10 and RAB8a/RAB39b for the subsequent formation of autophagosomes (Sellier et al., 2016; Sullivan et al., 2016; Yang et al., 2016). In addition, it was also observed that the C9orf72/SMCR8 heterodimer induced autophagy by phosphorylating ULK at Ser 757. Indeed, down-regulation of C9orf72 decreased the phosphorylation of ULK in MEF cells. Interestingly, this effect was reverted by SMCR8 overexpression, indicating that C9orf72-mediated ULK phosphorylation at Ser 757 is SMCR8 dependent (Yang et al., 2016). Altogether, these studies suggest that C9orf72 regulates autophagy initiation via ULK.

**Figure 6:**
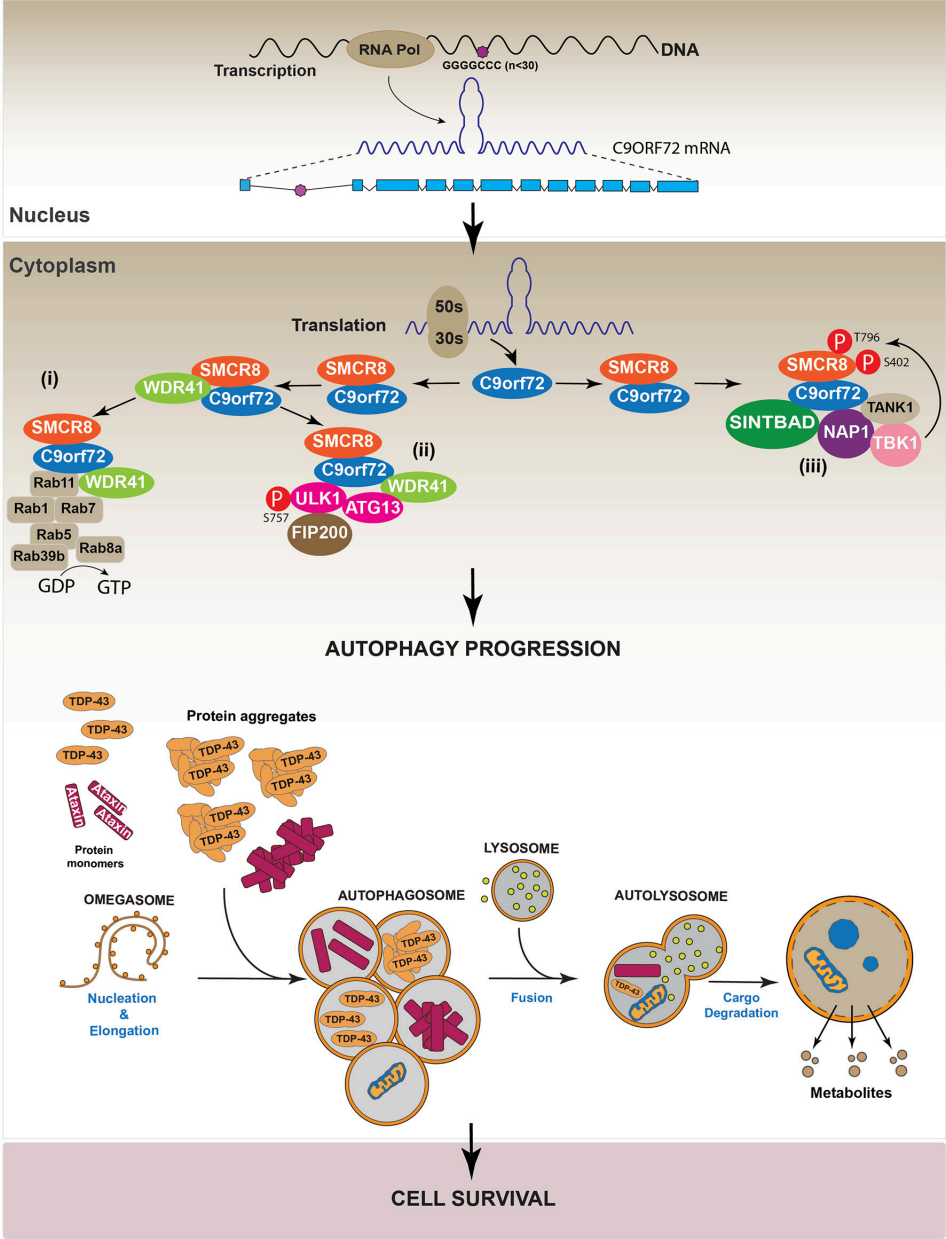
Regulation of autophagy by C9orf72. Studies indicate C9orf72 protein is implicated in autophagy regulation. Specifically, data show C9orf72 is involved in the progression of the autophagic process by three different mechanisms. (i) C9orf72 interacts with SMCR8 forming a heterodimer that recruits WDR4. The complex C9orf72/SMCR8/WDR41 activates several RAB proteins, which participate in autophagy. (ii) The C9orf72/SMCR8/WDR4 complex recruits and activates the ULK complex by its phosphorylation in S575. (iii) The heterodimer C9orf72/SMCR8 interacts with the proteins SINTBAD, NAP, and TANK. These proteins, in turn, recruit TBK1 to the complex allowing the phosphorylation of SMCR8, which is required for autophagy progression.

Additionally, data indicate that the C9orf72/SMCR8 complex also interacts with the TRAF Family Member Associated NFκB Activator protein (TANK), TBK1 Binding Protein 1 (SINTBAD) and Nucleosome Assembly Protein 1 (NAP1), forming a protein complex that activates the TANK-Binding Kinase 1 (TBK1). Active TBK1 phosphorylates SMCR8 at Ser 402 and Thr 796, stabilizing the C9orf72/SMCR8 complex and thus inducing autophagy. On the other hand, the interaction between C9orf72/SMCR8/WDR41 and ULK complex induced SMCR8 phosphorylation at Ser 400, 492, 562, and Thr 666/Ser 667. These modifications are less efficient in inducing autophagy compared with those performed by TBK1 (Sellier et al., 2016). Altogether, these studies indicate that C9orf72 is required to form a stable complex with SMCR8 and WDR41, thus allowing the recruitment of different factors such as the ULK complex and RABs to activate the autophagic machinery. The molecular mechanism supporting the new role of C9orf72 in autophagy regulation is summarized in Figure 6.

**Figure 7:**
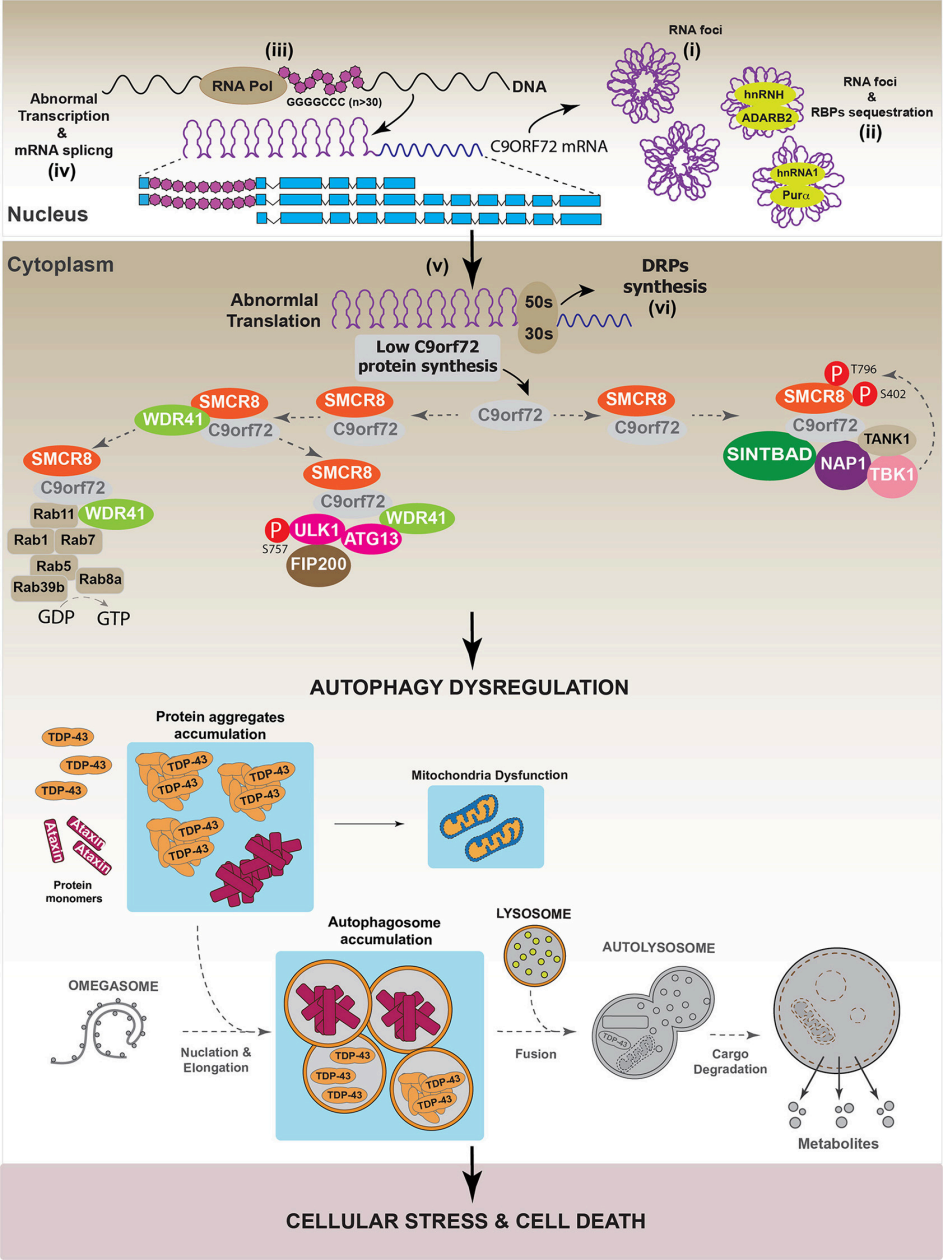
Autophagy dysregulation in C9orf72-dependent neurodegenerative diseases. In a pathological situation, it has been observed that the presence of GGGGCC repeats (*n* ≥ 30) in c9orf72 region can be toxic for the cell. Data indicate that GGGGCC repeats can internally interact to form RNA foci (i) that are toxic by recruiting different RNA binding proteins (RBPs) such as hnRNPH, hnRNPA2, ADARB2, and Purα. (ii) In addition to RNA foci, it has been shown that GGGGCC repeats interfere with the normal transcription (iii), splicing (iv), and translation (v) processes of C9orf72, thus reducing C9orf72 protein levels in the cell. Moreover, several studies showed that GGGGCC repeats can form polypeptides composed by dipeptides repetitions (DRPs). These DRPs are synthetized through a non-classical mechanism of protein translation (vi). To our knowledge, there is no evidence indicating that DRPs might interfere with the autophagic process. Overall, in pathological conditions, GGGGCC repeats decrease C9orf72 protein levels, thus affecting the normal autophagic process. Autophagy dysregulation culminates with the accumulation of autophagosomes, caused by impaired autophagosomal-lysosomal fusion, which might contribute to the formation and accumulation of protein aggregates like Ataxin and eventually TDP-43. This causes cellular stress and might lead to cell death. Gray color: represents down-regulated proteins and attenuated processes due to TDP-43 loss-of-function.

In agreement with the role of C9orf72 in autophagy, different works have demonstrated that C9orf72 down-regulation impaired autophagy progression, as evidenced by the intracellular accumulation of p62/SQSTM1 (Farg et al., 2014; Sellier et al., 2016; Yang et al., 2016). However, it is interesting to note that the dysregulation of the autophagic pathway induced by C9orf72 down-regulation did not induce cell toxicity (Sellier et al., 2016). However, in primary neuronal cultures and in a zebrafish *in vivo* model, the over-expression of Ataxin-2 Q30 aggregates induced cell toxicity when autophagy was inhibited by C9orf72 down-regulation (Sellier et al., 2016). Altogether, these results suggest that different pathways are implicated in the onset of C9orf72-mediated neurodegenerative diseases. One of these promotes the generation of aberrant protein inclusions, such as TDP-43 or Ataxin aggregates, while the second one involves the dysregulation of autophagy. To support this hypothesis, it has been observed that C9orf72 down-regulation increased TDP-43 aggregation in E8 mouse cortical neurons, indicating that the pathologies associated with reductions in C9orf72 level promote the onset of diseases characterized by the presence of TDP-43 aggregates (Sellier et al., 2016). Figure 7 summarizes the hypothetical role of C9orf72 in the onset of neurodegenerative diseases.

## Concluding remarks

Autophagy dysfunction has been identified in several neurodegenerative diseases such as ALS and FTLD. TDP-43 and C9orf72 are also associated with these illnesses as well as other neurodegenerative disorders. TDP-43 aggregates are found in neurons of ALS and FTLD patients, and it has been proposed that they may act as a sink for the newly synthetized TDP-43 protein, thus leading to TDP-43 loss-of-function. Autophagy has been proposed to be crucial for the maintenance of TDP-43 protein levels and to avoid TDP-43 aggregation. Indeed, very recently, data indicate TDP-43 is able to regulate autophagy by stabilizing the mRNA of important components of the autophagic machinery, and, conversely, TDP-43 down-regulation/loss-of-function finally inhibits the autophagic flux.

The association of C9orf72 with ALS and FTLD has recently been discovered, and the physiological functions of C9orf72 are still being investigated. Additionally, recent studies showed that C9orf72 is involved in membrane trafficking and autophagy. Specifically, it has been demonstrated that C9orf72 stimulates autophagy initiation by interacting with autophagic proteins involved in membrane trafficking. Consistently, in diseases such as ALS and FTLD, it has been observed a reduction in C9orf72 as well as decreased levels of autophagy.

Thus, to date, evidences strongly support that both, TDP-43 and C9orf72 regulate autophagy and that loss-of-function of any of these factors blocks the autophagic flux. In the scenario of TDP-43-associated proteinopathies, TDP-43 loss-of-function caused by TDP-43 aggregates, can further increase cellular stress by blocking the autophagic process. In turn, autophagy inhibition provokes an exacerbated accumulation of TDP-43 aggregates, enhances cellular stress and induces cell death, leading to the onset or progression of neurodegenerative diseases.

Consistently, in C9orf72-associated neuropathologies, two events lead to the onset of the disease. The first one is associated with the production of aberrant protein inclusions, such as TDP-43 or Ataxin aggregates, while the second one is characterized by the deregulation of autophagy caused by C9orf72 loss-of-function.

Thus, the dysregulation of autophagy is key in the development of TDP-43- and C9orf72-associated neurodegenerative disorders. Even though different are the mechanisms that cause the impairment in autophagy, induction in the autophagic pathway can be considered as a general strategy to avoid these diseases, and research should focus on the identification of novel therapeutic strategies to stimulate autophagy.

## Author contributions

MB, EM, and AC wrote the article and made the figures. EB made important contributions, discussions and corrections to the manuscript.

### Conflict of interest statement

The authors declare that the research was conducted in the absence of any commercial or financial relationships that could be construed as a potential conflict of interest.

## Footnotes

^1^http://www.un.org/en/development/desa/population/publications/pdf/ageing/WPA2015_Report.pdf
